# Identification of a novel polymorphism associated with reduced clozapine concentration in schizophrenia patients—a genome-wide association study adjusting for smoking habits

**DOI:** 10.1038/s41398-020-00888-1

**Published:** 2020-06-19

**Authors:** Robert Løvsletten Smith, Kevin O’Connell, Lavinia Athanasiu, Srdjan Djurovic, Marianne Kristiansen Kringen, Ole A. Andreassen, Espen Molden

**Affiliations:** 1 Center for Psychopharmacology, Diakonhjemmet Hospital, Oslo, Norway; 2 CoE NORMENT, Division of Mental Health and Addiction, Oslo University Hospital, and Institute of Clinical Medicine, University of Oslo, Oslo, Norway; 3 Department of Medical Genetics, Oslo University Hospital, Oslo, Norway; 4 NORMENT, Department of Clinical Science, University of Bergen, Bergen, Norway; 5 Department of Life Sciences and Health, Oslo Metropolitan University, Oslo, Norway; 6 Department of Pharmacy, Section for Pharmacology and Pharmaceutical Biosciences, University of Oslo, Oslo, Norway

**Keywords:** Pharmacogenomics, Schizophrenia

## Abstract

Clozapine (CLZ) is the superior antipsychotic drug for treatment of schizophrenia, but exhibits an extensive interpatient pharmacokinetic variability. Here, we conducted a genome-wide association study (GWAS) of CLZ serum concentration adjusting for known smoking habits, which is a major nongenetic factor reducing CLZ levels. The study included 484 patients with 10,283 steady-state serum concentrations of CLZ and *N*-desmethylclozapine, prescribed dosing, co-medications and known smoking habits (*n* = 422; 9284 serum samples) from a therapeutic drug monitoring (TDM) service. The GWAS analyses were performed with and without smoking habits as covariate, where possible hits were assessed in relation to the target CLZ concentration range applied in the TDM service (300–2500 nmol/L). The smoking-independent analysis of *N*-desmethylclozapine serum concentration and the CLZ-to-*N*-desmethylclozapine ratio replicated the previously identified locus on chromosome 4. After adjusting for smoking habits in patients confirmed as ‘smokers’ (61%) or ‘nonsmokers’ (39%), a novel variant (*rs28379954*; minor *T*>*C* allele frequency 4.1%; 7.6% *CT* carriers in the population) within the gene encoding the nuclear factor 1 B-type (*NFIB*) was significantly associated with reduced CLZ serum concentration (*p* = 1.68 × 10^−8^, beta = −0.376; explained variance 7.63%). There was no significant association between *rs28379954* and *N*-desmethylclozapine concentration in the GWAS analysis (*p* = 5.63 × 10^−5^). The fraction of CLZ TDM samples below 300 nmol/L was significantly higher in carriers vs. noncarriers of the *rs28379954* minor *C* allele [12.0% (95% CI: 9.4–14.7) vs. 6.2% (95% CI: 5.7–6.8), *p* < 0.001]. We identified a novel variant in the *NFIB* gene associated with reduced CLZ levels and increased risk of subtherapeutic serum concentrations. This warrants testing of clinical relevance of screening for this gene variant, and also experimental studies to investigate the biological mechanisms of NFIB involvement in CLZ pharmacokinetics.

## Introduction

Clozapine (CLZ) is the most efficient antipsychotic drug in treatment of schizophrenia^1^. However, CLZ is only indicated in treatment-resistant schizophrenia (TRS) due to risk of neutropenia or agranulocytosis^2,3^, which is reported to occur in about 3% and 0.5–1% of the patients, respectively^4^. Therefore, close monitoring of neutrophil granulocyte counts is mandatory during use of CLZ^5^. The suppression of granulocyte levels, as well as risk of other adverse effects such as seizures, cardiometabolics (weight gain and type 2 diabetes), sedation, and hypersalivation, have been associated with CLZ and/or metabolite levels^6,7^.

CLZ is subjected to complex metabolism involving biotransformation via multiple pathways^8,9^. Several cytochrome P450 (CYP) enzymes, including CYP1A2, CYP3A4, CYP2D6, and CYP2C19, are in vitro shown to be involved in the oxidative metabolism of CLZ to the major metabolites *N*-desmethylclozapine and clozapine-*N*-oxide^9^. In addition, CLZ undergoes metabolism to reactive nitrenium ion metabolites, which are believed to be important for the toxicity of CLZ treatment^10,11^. Less is known about the enzyme(s) mediating the formation of nitrenium ion metabolites of CLZ, but the myeloperoxidase system in granulocytes seem to play a role^9,12^.

Previous studies have shown that interindividual variability in CLZ concentration is significantly associated with age, sex, and smoking habits^13,14^, where the latter is quantitatively most important. However, these factors only explain parts of the variability, which has motivated the search for pharmacogenetic determinants of CLZ pharmacokinetics. Most studies so far have been unsuccessful in identifying genetic polymorphisms associated with individual variability in CLZ pharmacokinetics^15^, but recently a genome-wide association study (GWAS) in a British patient population (*n* = 2989) identified a single locus (15:74817689–75404506) between the *CYP1A1* and *CYP1A2* genes associated with a reduction in CLZ serum levels^16^. Further, a locus on chromosome 4 (4:69542100–70387482), containing the *UGT2B10* gene, was associated with both *N*-desmethylclozapine serum concentration and the CLZ-to-*N-*desmethylclozapine metabolic ratio. For the *N*-desmethylclozapine serum concentration and CLZ-to-*N*-desmethylclozapine metabolic ratio two additional loci were identified^16^, i.e., at chr2:234611523–234676118 and 10:96098093–96974830, respectively. In contrast to polygenic complex traits, such as schizophrenia^17^, these results support the hypothesis that the genetic architecture of CLZ metabolism is less polygenic and may constitute a smaller number of variants with larger effect sizes^16,18^. This is typical for the pharmacogenetics of drug-metabolizing enzymes, where a limited number of variant allele diplotypes have large effect sizes and separate the population into distinct metabolizer phenotypes^19,20^. In addition, CLZ related adverse effects are associated with few genetic variants with large effect sizes^21,22^.

Tobacco smoke reduces the serum concentration of CLZ by ~30–50% due to polycyclic aromatic hydrocarbons inducing gene transcription of drug-metabolizing enzymes, e.g., CYP1A enzymes^23–25^. Thus, information on smoking habits is crucial when studying factors that may influence the CLZ serum concentrations. The previous British GWAS did not account for the individual patient’s smoking habits^16^, but used a proxy measure (polygenic risk scores for smoking), which may imply that significant variants were not captured in the analysis. The aim of the present study was to perform a GWAS on serum concentrations of CLZ and its main metabolite *N*-desmethylclozapine in a Norwegian population of schizophrenia patients with known smoking habits.

## Materials and methods

### Study population

Patients with serum concentration measurements of CLZ and the metabolite *N*-desmethylclozapine, and biobanked blood samples for genotyping, were included from the therapeutic drug monitoring (TDM) service at the Center for Psychopharmacology, Diakonhjemmet Hospital, Oslo, Norway, from March 2005 to May 2019. Inclusion criteria were: (i) blood sampling for serum concentration analysis of CLZ at steady-state conditions and 10–30 h after the last drug intake, and (ii) prescribed daily dose of CLZ. For the GWAS analysis adjusting for smoking only, a further inclusion criterion was (iii) information about confirmed smoking habits written by the physician on the TDM requisition form (cigarette smoking ‘yes’ or ‘no’). The time intervals between last dose intake and blood sampling for serum concentration analysis, and the psychotropic co-medication profiles, were obtained from the requisition forms. Exclusion criteria were (i) concurrent use of interacting drugs, i.e., the CYP1A2/2C19/3A4 inhibitor fluvoxamine, or the CYP3A4/2C19 inducers phenobarbital, phenytoin, and carbamazepine, and (ii) concentration of CLZ or *N*-desmethylclozapine below the lower limit of quantification (LLOQ; i.e., 20 nmol/L). All serum concentration measurements from each of the patients fulfilling the criteria were included in the statistical analyses, as described below.

The study was approved by the Regional Committee for Medical and Health Research Ethics and the Hospital Investigational Review Board. As the study was solely based on historical data, the project did not have the potential to cause any harm or burden, informed patient consent was not required.

### Clinical characteristics

The study population consisted of patients with TRS as determined by TDM of CLZ per se, since use of this drug is solely indicated for TRS^3^. In Norway, TDM analyses are reimbursed by the governmental health service, and it is used as a tool for clinical follow-up in psychiatry. The analyses are typically requested as a routine control to monitor e.g., adherence and drug–drug interactions, or to assess adverse effects or lack of effect, in relation to a therapeutic concentration reference range. All results from the analyses are interpreted by the TDM laboratory against the therapeutic reference range as basis for the reports sent to the physicians.

The therapeutic reference ranges used in our TDM service are based on international standards as defined by the AGNP working group^26^. However, they are adjusted according to the concentrations measured at our laboratory in a Norwegian patient population during therapeutic dosing. In the case of CLZ, the applied therapeutic reference range at our laboratory is 300–2500 nmol/L, while the AGNP reference range is 1071–1835 nmol/L. The AGNP definition of 1071 nmol/L (=350 ng/ml) as the lower therapeutic concentration boundary is based on several studies correlating clinical response and serum concentration of CLZ, e.g., the study by Perry et al. showing that only 22% of the patients with concentration <350 ng/mL were responders (i.e., ≥20% reduction in symptom score), while 64% of the patients above the limit were responders^27^. This was supported by a follow-up study where the majority of nonresponders achieved clinical effects when the serum CLZ levels were raised above 350 ng/mL^28^. However, in the present study, we use a more conservative lower serum concentration boundary, i.e., 300 nmol/L, since it is applied in our clinical TDM service based on Norwegian data. Use of 300 nmol/L as the lower boundary also increases the likelihood of poor clinical efficacy of CLZ when serum concentrations are below this threshold. In addition, a CLZ concentration of 300 nmol/L corresponds with a brain dopamine-2 (D2) receptor occupancy of about 20–40%, which is regarded as critical for clinical effect^29^.

The TDM service at the Diakonhjemmet Hospital in Oslo analyses serum samples from psychiatric patients mainly located in the South-Eastern Norway Regional Health Authority, and ~25% of the submitted samples are from hospitalized patients.

### Genotyping and imputation

DNA was extracted from whole blood and genotyped with the Human Omni Express-24 v.1.1 array (Illumina Inc., San Diego, CA, USA), at deCODE Genetics (Reykjavik, Iceland), in accordance with the standard Illumina protocol. Standard pre-imputation quality control was performed using PLINK v1.9^30,31^. Finally, chromosome-wide haplotypes were phased with Eagle2^32,33^ and missing variants were imputed with Minimac3^34^ using the first release of the haplotype reference consortium reference set. After imputation, we removed variants if their minor allele frequency was <1% or if they showed departure from Hardy–Weinberg equilibrium (*P*-value <1 × 10^−6^). Furthermore, we removed related individuals (pairwise Identity-By-Descent $\hat \pi$ > 0.2 according to PLINK v1.9^30,31^) and those individuals with high rates of genotyping missingness (>5%). The final dataset included 484 unrelated individuals and ~6.2 million variants for analysis.

### GWAS of serum concentrations with and without adjusting for smoking habits

We applied a regression modeling framework to combine serum concentration measurements of each patient from multiple time points into a single phenotype, as described previously^16^. The R package fitdistrplus v1.0-14 was used to determine the best-fitting distribution for each phenotype outcome variable (dose-adjusted serum concentrations of CLZ and *N-*desmethylclozapine, and the CLZ-to-*N-*desmethylclozapine metabolic ratio). Thereafter, the R package gamlss v5.1-2 was used to specify a random-effects model for each phenotype outcome variable. The fixed effect covariates in the model included known predictors of CLZ serum concentration (CLZ dose, age at sampling and time between sampling and last dose intake). In addition, a random effect was added to model the distribution of the outcome in each individual, controlling for these covariates. The inclusion of this random effect for each individual permits unbalanced data patterns (differing number of serum measurements) in the model^35^. For each model, the coefficients of this random effect for each individual were extracted. These coefficients correspond to the variation within the serum concentrations for each individual independent of the included covariates, and were used as the outcome phenotypes for the GWAS analyses.

We conducted a GWAS for each outcome phenotype (variation in serum concentrations of CLZ and *N*-desmethylclozapine, and the CLZ-to-*N*-desmethylclozapine metabolic ratio) using linear regression analyses implemented in PLINK v1.9^30,31^, controlling for participant age, sex, the first 20 genetic principal components, and genotyping batch. We also performed a GWAS for each outcome phenotype in the sample with known smoking habits (*n* = 422) by adding this variable as a covariate in the regression models.

Details describing the methods used for gene loci definitions and annotations, estimating the single-nucleotide polymorphism (SNP) proportion of variance explained, and follow-up GWAS meta-analyses with the British sample including testing the effect of smoking PRS as proxy for smoking and body mass index, are included in the Supplementary file (Supplementary Methods; Supplementary Figs. 1–4 and Supplementary Tables 1 and 2). Also included is a methodological description of the targeted Taqman genotyping used for verification of SNPs significantly associated with CLZ concentration in the GWAS.

### Follow-up analyses of SNPs identified in the GWAS

For SNPs significantly associated with CLZ concentration in the GWAS, the various phenotypes were compared in carriers vs. noncarriers of the identified variant(s) using linear mixed model analysis. These analyses were performed for both smokers and nonsmokers adjusting for sex, age, and sampling time as covariates. The Pearson Chi-square test was used to evaluate the potential differences in proportion of patients with TDM measurements of CLZ either below or above the defined target concentration range (300–2500 nmol/L; c.f. information provided in the beginning of the “Methods” section).

### Statistical tools

The statistical analyses were performed using R statistics and PLINK v1.9^30,31^ for the GWAS and SPSS^®^ (version 25.0) for the assessments of the significant GWAS hits, as described above.

## Results

### Sample characteristics

The study population comprised 484 CLZ-treated patients with a total of 10,283 CLZ and *N*-desmethylclozapine serum concentrations measurements (Table 1), with median age of 37 years (IQR: 29–47) and 38.4% females (*n* = 186). The median prescribed daily dose of CLZ was 400 mg (IQR: 250–500 mg), whereas the median serum concentration of CLZ and *N*-desmethylclozapine were 1167 and 770 nmol/L, respectively. Among the patients with known smoking habits (*n* = 422; 9284 serum samples), 258 were confirmed smokers (61.1%) and 164 were confirmed nonsmokers (38.9%). The median dose-adjusted CLZ serum concentrations (C/D ratio) were 2.46 nmol/L/mg (IQR: 1.67–3.64) and 4.63 nmol/L/mg (IQR: 2.91–6.49) in smokers and nonsmokers, respectively, whereas the median CLZ-to-*N*-desmethylclozapine ratios were 1.43 (IQR: 1.17–1.71) in smokers and 1.69 (IQR: 1.40–2.06) in nonsmokers.

**Table 1:** Population characteristics.

Variables	Value
No. of patients, *n* (measurements)	484 (10,283)
Male/female, *n*	298/186
Age, years; median (IQR)	37 (29, 47)
No. of measurements per patient; median (IQR)	13 (5, 28)
Clozapine concentration, nmol/L; median (IQR)	1167 (687, 1786)
*N*-desmethylclozapine concentration, nmol/L; median (IQR)	770 (469, 1135)
Clozapine dose, mg (IQR)	400 (250, 500)
Withdrawal time (time between dose and sampling), *h*, median (IQR)	13.1 (12.2, 14.5)
Smoking habits, *n* (%)	422 (87.2)
Smoker/nonsmoker (% females)	258 (31.4)/164 (48.2)

### GWAS of serum concentrations adjusting for smoking habits

**Fig. 1:**
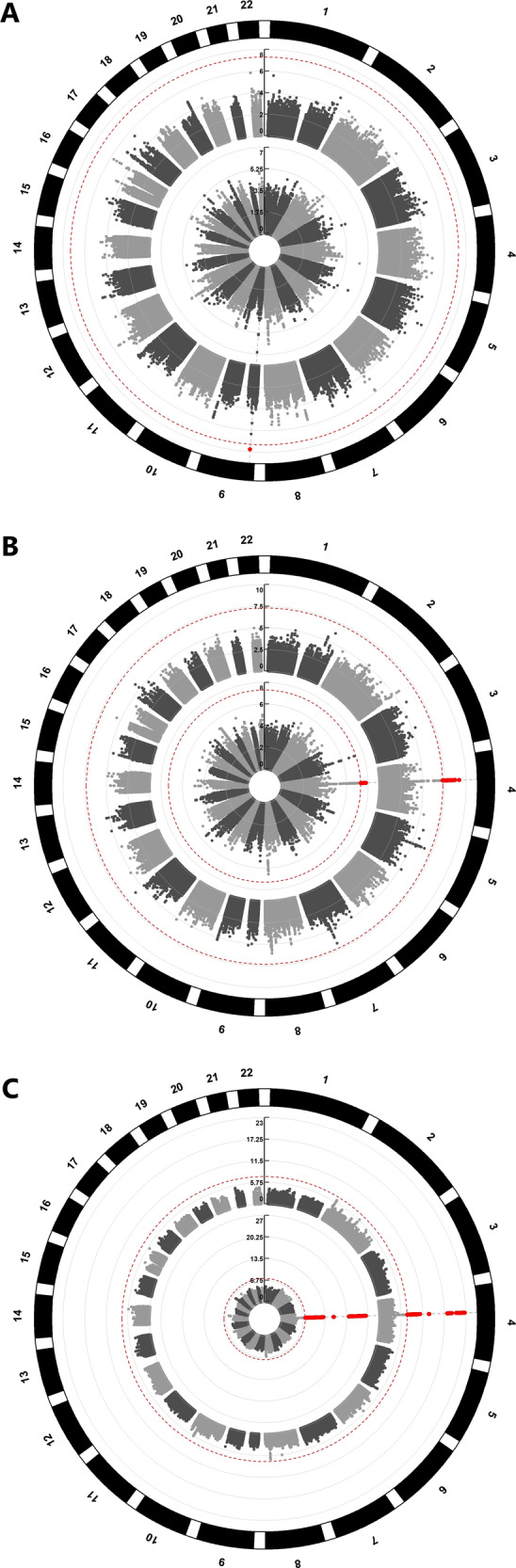
Manhattan plots showing clozapine concentration, *N*-desmethylclozapine concentration, and metabolic ratio associations. Circular Manhattan plots for (A) clozapine serum concentration, (B) *N*-desmethylclozapine serum concentration, and (C) Clozapine-*N*-desmethylclozapine metabolic ratio. The inner plot shows the results from the initial GWAS without controlling for smoking habits. The outer plot shows the results of the GWAS after controlling for smoking habits. The −log10 transformed *p*-values for each SNP are indicated on the *y*-axes and chromosomal positions along the exterior of the plot. The dashed red line represents the threshold for significant associations (*p*<5×10^−8^). SNPs surpassing this threshold are indicated in red.

The GWAS of CLZ serum concentration, adjusting for smoking habits (*n* = 422), identified a single novel genome-wide significant locus at chromosome 9 (Fig. 1a, Table 2 and Supplementary Fig. 5a), represented by lead SNP (*rs28379954*, *p* = 1.677 × 10^−8^, 9:14163907). Data from the 1000 Genomes phase 3-genome browser shows that this SNP lies within an LD-sparse region (Supplementary Fig. 6). However, four other SNPs, in LD with the lead SNP (*r*^2^ > 0.2) and within 250 kb, showed nominal significance (*p* < 1.000 × 10^−4^): *rs10481501 C*>*T* (9:14172871, *p* = 4.506 × 10^−7^, LD *r*^2^ = 0.554), *rs10961406 A*>*G* (9:14182043, *p* = 3.856 × 10^−5^, LD *r*^2^ = 0.451), *rs10120632 G*>*C* (9:14226937, *p* = 3.892 × 10^−5^, LD *r*^2^ = 0.329) and *rs12156582 C*>*T* (9:14163383, *p* = 4.546 × 10^−6^, LD *r*^2^ = 0.411; Supplementary Fig. 7).

**Table 2:** Association statistics and effect sizes of the lead SNPs for each phenotype and genomic locus from the GWASs, without or with controlling for smoking habits.

Phenotype and locus	Lead SNP	Position: alleles	Effect allele	GWAS p	Beta	SE	Annotation	Nearest gene	Distance to gene	CADD	RDB	Min Chr state	Common Chr state
Statistics without controlling for smoking habits:													
*N*-desmethylclozapine:													
4:69601886–70138176	rs1670747	69610466: G/A	A	1.616 × 10^−^^8^	−0.183	0.032	Intergenic	*RP11-1267H10.4*	32249	2.713	NA	2	15
Metabolic Ratio:													
4:69535335–70387482	rs1513559	69655555: A/G	G	4.819 × 10^−^^27^	0.246	0.021	Intergenic	*CTD-2005D20.1*	2362	0.004	5	9	15
Statistics controlling for smoking habits:													
Clozapine:													
9:14163907–14163907	rs28379954	14163907: T/C	C	1.677 × 10^−^^8^	−0.376	0.065	Intronic	*NFIB*	0	8.433	7	5	15
*N*-desmethylclozapine:													
4:69601886–70138176	rs1670747	69626729: G/A	A	5.805 × 10^−^^10^	−0.215	0.034	Intergenic	*RP11-1267H10.4*	32249	2.713	NA	2	15
Metabolic ratio:													
4:69544277–70340670	rs292638	69662832: C/G	G	2.067 × 10^−^^23^	0.247	0.023	Intergenic	*CTD-2005D20.1*	9639	2.223	5	5	15

CADD = Combined Annotation-Dependent depletion score, which predict how deleterious the SNP effect is on protein structure/function (higher scores indicate more deleterious); RegulomeDB (RDB) scores predict likelihood of regulatory functionality (lower scores indicate higher likelihood); min Chr State = minimum chromatin state across 127 tissue types (lower scores indicate more open chromatin); common Chr State = most common chromatin state in 127 tissue types.

The *rs28379954* variant is located within an intron of the nuclear factor 1 B-type (*NFIB*) gene and the minor *C* allele (*C* allele frequency = 0.041; explained variance 7.63%) was associated with reduced CLZ serum concentration (beta = −0.3755; Table 2 and Fig. 1a). The *NFIB* genotypes from GWAS and targeted Taqman analyses of *rs28379954 T*>*C* showed 97% consistency, supporting the significant association with CLZ serum concentration. Functional analyses do not suggest this SNP, or the four others in LD (*r*^2^ > 0.2), to be deleterious (CADD scores: <12.37) or likely to have regulatory functionality (RegulomeDB scores = 5–7). Furthermore, none of these SNPs were associated with any other phenotype in the GWAS catalog and none were identified as potential eQTLs in the GTEx portal.

GWAS analyses of *N-*desmethylclozapine serum concentration and the CLZ-to-*N*-desmethylclozapine ratio as phenotypes, adjusting for smoking habits, revealed the same significant loci as those identified in the GWAS analyses by Pardiñas et al.^16^. (Fig. 1b, c, Table 2, and Supplementary Fig. 5b, c). We did not replicate the additional locus associated with *N*-desmethylclozapine serum concentration in the study by Pardiñas et al.^16^ (2:234611523–234676118). Of the 242 SNPs within this region, the most significant association to *N*-desmethylclozapine concentration was identified for *rs17862876* (*p* = 0.059, beta = −0.227). Furthermore, we did not replicate the additional locus associated with the CLZ-to-*N*-desmethylclozapine ratio (10:96098093–96974830). Of the 2098 SNPs within this region, the most significant association to the CLZ-to-*N*-desmethylclozapine ratio was identified for *rs66634733* (*p* = 1.065 × 10^−4^, beta = 0.80). Functional annotation of the lead SNPs from these GWAS did not predict any deleterious effects on protein structure/function and did not suggest any regulatory functionality (Table 2).

**Fig. 2:**
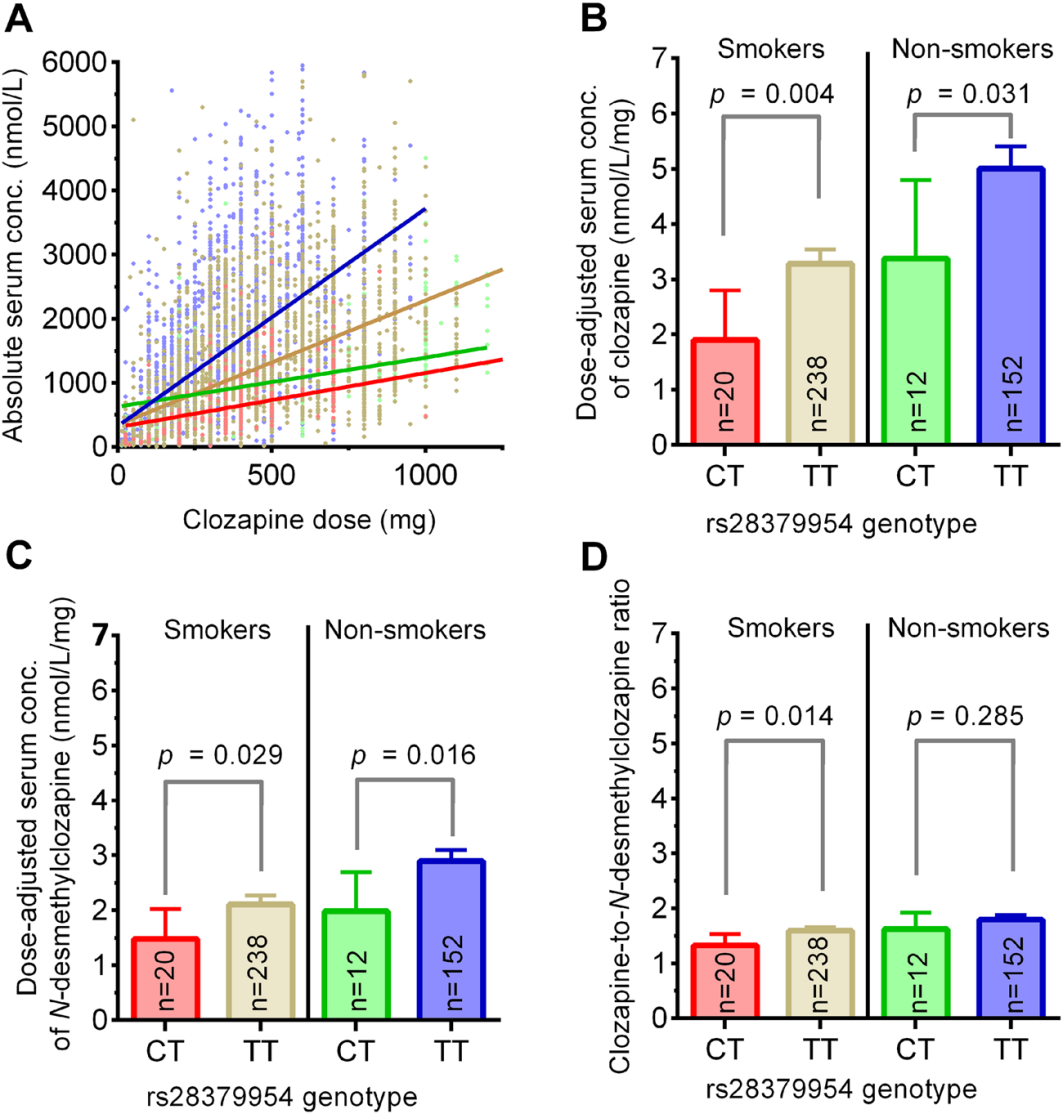
The impact of *NFIB rs28379954* minor *C* allele carriers on clozapine and *N*-desmethylclozapine concentration, as well as clozapine-to-*N*-desmethylclozapine ratio, in relation to smoking habits. **a** The figure shows all serum concentration measurements (dots) versus clozapine prescribed daily dose. The regression lines for the various subpopulations are calculated using a linear mixed model analyses without any covariates (blue, nonsmoking *rs28379954 TT* carriers; golden, smoking *TT* carriers; green, nonsmoking *CT* carriers; red, smoking *CT* carriers). The figures show the impact of *rs28379954* minor *C* allele carriers on dose-adjusted serum concentration of clozapine (C/D ratio; **b**), *N*-desmethylclozapine (C/D ratio; **c**) and clozapine-to-*N*-desmethylclozapine ratio (**d**) in relation to smoking habits. The ratios were analyzed by linear mixed model including sex, age, and sampling time (time between last dose and blood sampling) as covariates. The numbers of patients within each subpopulation are shown in the various bars.

Among the patients with smoking habits data (*n* = 422), 32 patients (7.6%) were in the GWAS identified as *rs28379954* minor *C* allele carriers. In Fig. 2a, the linear regressions of absolute, unadjusted CLZ serum concentration vs. prescribed daily dose are shown according to *rs28379954* genotype. As compared with the *rs28379954 TT* genotype carriers (smoker*s*: 2.31 (95% CI: 2.19–2.44); nonsmokers: 3.39 (95% CI: 3.15–3.64)), the *C* allele carriers (smokers: 0.81 (95% CI: 0.56–1.06); nonsmokers: 0.77 (95% CI: 0.37–1.16)) had a significantly lower linear slope (Fig. 2a). This was reflected by a significantly higher proportion of CLZ serum concentrations below the therapeutic range (<300 nmol/L) in *C* vs. *TT* allele-carrying smokers (16.0% vs. 7.2%, *p* < 0.001) and nonsmokers (8.0% vs. 4.9%, *p* = 0.017), despite similar daily dosing (*p* > 0.05). Regarding the pharmacokinetic effect of *rs28379954, C* allele carriers had an ~42% lower CLZ dose-adjusted serum concentration than *TT* genotype carriers in smokers (*p* = 0.004), whereas the *C* allele-carrying nonsmokers had 33% lower CLZ dose-adjusted serum concentration ratio than the *TT* genotype carrying nonsmokers (*p* = 0.031; Fig. 2b), after adjusting for age, sex, and sampling time as covariates. The dose-adjusted serum concentration of *N*-desmethylclozapine and CLZ-to-*N*-desmethylclozapine ratio were not associated with the *rs28379954 C*>T polymorphism in the GWAS analyses (Table 2). Due to reduced substrate (CLZ) availability, the *rs28379954 C* allele carriers still had 30% and 31% lower dose-adjusted serum concentration of *N*-desmethylclozapine compared with the smoking (*p* = 0.029) and nonsmoking (*p* = 0.016) *TT* genotype carriers, respectively (Fig. 2c). In smoking *rs28379954 TT* allele carriers, the CLZ-to-*N*-desmethylclozapine ratio was 19% (*p* = 0.014) higher than in smoking *C* allele carriers (Fig. 2d), while no significant change in CLZ-to-desmethylclozapine ratio was observed among *C* allele-carrying nonsmokers (*p* = 0.265; Fig. 2d).

### GWAS of serum concentrations without adjusting for smoking habits

Without adjusting for smoking habits, we identified no genome-wide significant loci in our GWAS of CLZ serum concentration (Fig. 1a and Supplementary Fig. 8a). Further investigation of the previously implicated *CYP1A* locus on chromosome 15 (15:74817689–75404506)^16^ showed that the lead SNP *rs2472297* had a call rate of 64.2% and was therefore excluded from our GWAS analyses. However, 773 SNPs were present within this locus, and the most significant association was observed for *rs11635266 T*>*C* (15:75386649) at *p* = 0.002 (beta = 0.080).

Our analysis of *N*-desmethylclozapine serum concentration and the CLZ-to-*N*-desmethylclozapine ratio replicated the significant locus on chromosome 4 (Fig. 1b, c, Table 2 and Supplementary Fig. 8b, c) reported by Pardiñas et al.^16^, regardless of adjusting for smoking habits (*p* < 1.62 × 10^−8^). On the other hand, we did not replicate the additional loci previously associated with *N*-desmethylclozapine serum concentration (2:234611523–234676118) or with the CLZ-to-*N*-desmethylclozapine ratio (10:96098093–96974830), respectively.

Meta-analyses of our smoking-independent GWAS and the previously published British GWAS^16^ did not identify any novel significant loci, while all previously identified loci were retained (Supplementary Figs. 1 and 2, Supplementary Table 1).

## Discussion

The present study replicated the previously identified locus on chromosome 4 for both *N-*desmethylclozapine and the CLZ-to-*N-*desmethylclozapine metabolic ratio, independent of smoking habits^9,24^. When adjusting for known smoking habits, which have a major effect on CLZ metabolism^13,14^, we identified a novel locus in the *NFIB* gene (*rs28379954 T*>*C*) associated with a significant reduction in CLZ serum concentrations.

According to our findings in the GWAS analysis adjusting for smoking habits, heterozygous carriers of the minor *rs28379954 C* allele are associated with a substantial decrease in CLZ serum concentration, i.e., an estimated reduction of 37.6% in the dose-adjusted serum concentration of CLZ compared with homozygous carriers of the wild type allele (*rs28379954 TT*). This effect was only identified after adjusting for individually ascertained smoking habits, and not when controlling for a polygenic score proxy measure of smoking, as performed in the recent GWAS by Pardiñas et al.^16^. Smokers comprised 61.1% of the included patient population, a typical prevalence for a sample of individuals with schizophrenia^36^. These results suggest that patients heterozygous for the minor *C* allele, representing 7.6% of the current study population, are at risk of subtherapeutic CLZ serum concentrations at standard recommended CLZ dosing. Indeed, the patients carrying the minor *C* allele had significant lower absolute CLZ serum concentration regardless of their smoking habits; however, smoking was shown to reinforce this effect. Since smokers are already prone to lower CLZ serum concentrations, smokers carrying the minor *C* allele may represent a subpopulation particularly vulnerable to suboptimal concentration and treatment failure. This is supported by the more than threefold higher proportion of CLZ concentration measurements below the therapeutic reference in smoking *C* carriers vs. nonsmoking *TT* carriers in our population. Thus, genotyping of *rs28379954* may potentially aid individualized dosing of CLZ in clinical practice to ensure optimal treatment response.

The *rs28379954 T*>*C* polymorphism was not associated with the pharmacokinetic variability of either the dose-adjusted serum concentration of *N*-desmethylclozapine or the CLZ-to-*N*-desmethylclozapine metabolic ratio in the GWAS analyses, suggesting that the mechanisms behind the reduced CLZ levels may be related to an increased formation of other CLZ metabolites than *N-*desmethylclozapine. The SNP *rs28379954* is located in an intronic region of the *NFIB* gene, which encodes a transcription factor (NFIB) that is expressed in several tissues, such as lungs, liver, kidneys, and brain^37,38^. NFIB plays a role during embryotic development by initiating tissue differentiation in the fetus^38^, but has not previously been reported to regulate the expression or activity of drug-metabolizing enzymes.

From the findings of the present study, it may be hypothesized that the *rs28379954 T*>*C* polymorphism affects the transcription of genes involved in CLZ metabolism. Based on observations of a quantitatively higher and statistically stronger difference in CLZ levels in smoking than nonsmoking *CT* vs. *TT* carriers (Fig. 2b), the most relevant hypothesis is that *NFIB* modulates the expression of the ligand-activated transcription factor aryl hydrocarbon receptor (AhR), which is induced by smoking and important for CLZ metabolism. However, experimental studies are required to elucidate the functional impact of the *NFIB* gene on CLZ metabolism. In addition, it should be investigated if *rs28379954* genotype is associated with formation of metabolites with potential impact on CLZ toxicity.

Recently, Pardiñas et al. showed an association between the SNP *rs2472297* in *CYP1A1/1A2* locus and CLZ serum concentration in a British cohort^16^, which was not replicated in the present study. In their study, however, smoking habits data were not available, and polygenic risk scores for smoking were therefore used as a proxy measure of smoking habits instead. Notably, cigarette smoking has been shown to reduce CLZ levels by ~30–50% by inducing CYP1A1/1A2^23–25^, and up to 60% of individuals with schizophrenia are smokers^36^. Given the large effect of smoking on CLZ metabolism, and the high prevalence of smoking in people with schizophrenia, the differences in accounting for smoking habits may explain the discrepancy between the studies. It should be noted that in the present study, the SNP *rs2472297* in the *CYP1A1/1A2* locus had a call rate of 64.2% and was therefore excluded from GWAS analyses. However, no other SNPs within the previously defined *CYP1A1/1A2* locus (15:74817689–75404506) showed association signals, without and after adjusting for smoking habits, respectively. These results suggest that caution is required when polygenic risk score is utilized as a proxy for smoking.

Similarly to Pardiñas et al.^16^, we found that the genetic variants in the *UGT2B10* locus are associated with the *N*-desmethylclozapine serum concentration, and hence the CLZ-to-*N*-desmethylclozapine metabolic ratio, irrespective of smoking habits. In particular, the intergenic minor alleles of the lead SNPs *rs1670747* (4:69601886–70138176) and *rs2926038* (4:69544277–70340670) are responsible for decreasing *N*-desmethylclozapine levels and increasing the CLZ-to-*N*-desmethylclozapine ratio by ~21% and 25%, respectively. UGT2B10 is involved in *N*-glucuronidation of a variety of psychotropic drugs including CLZ, and likely *N*-desmethylclozapine^39^. These variants might be of potential relevance to genotype before starting CLZ treatment, since increasing *N*-desmethylclozapine level has been associated with positive effects of CLZ treatment^6,40^, but further studies are required to elucidate if *UGT2B10* genotype has an impact on the clinical response of CLZ.

Our results add support to the hypothesis that the genetic architecture of CLZ metabolism is driven by few variants with large effect sizes^16,18^, in line with typical pharmacogenetics findings^19,20^. Despite our sample being approximately sixfold smaller than the British GWAS^16^, we robustly replicate their findings that the *UGT2B10* locus on chromosome 4 is associated with *N*-desmethylclozapine serum concentration, and hence the CLZ-to-*N*-desmethylclozapine metabolic ratio. These results, in addition to the 97% overlap in *rs28379954 T*>*C* genotypes by GWAS and targeted Taqman analyses, increase our confidence that the novel locus we identified to be associated with CLZ serum concentration (*rs28379954*), after adjusting for smoking habits, is a true positive and not a spurious association due to winner’s curse. Additional studies are still required to replicate this association in independent cohorts of CLZ-treated schizophrenia patients with known smoking habits.

The use of TDM data for research purposes is associated with some methodological limitations, where potential nonadherence is a relevant issue creating ‘nonbiological’ variability in the concentration phenotype. Unknown co-medications may also alter CLZ metabolism and concentration, but in the current material, co-prescription of the most relevant interacting drugs was accounted for, i.e., phenytoin, phenobarbital, carbamazepine, and fluvoxamine, which all change the CLZ concentration several-fold^41,42^. In addition, information on comorbidity, weight, and potential consumption of dietary supplements potentially affecting concentration phenotypes was not available. The amount of cigarette consumption could not be obtained, but the enzyme-inducing effect of smoking is little affected by the degree of cigarette consumption^25^.

In conclusion, the present GWAS on CLZ pharmacokinetics, adjusting for known smoking habits, identified a novel variant in the *NFIB* gene (*rs28379954*) associated with a significant reduction in CLZ levels. These results suggest that patients carrying the minor *C* allele are at risk of subtherapeutic CLZ serum concentrations, particularly in smokers. Thus, genotyping of *rs28379954* may potentially aid individualized dosing of CLZ in clinical practice.

## Supplementary information

Supplementary methods

Supplementary figure and table legends

Supplementary figure 1

Supplementary figure 2

Supplementary figure 3

Supplementary figure 4

Supplementary figure 5

Supplementary figure 6

Supplementary figure 7

Supplementary figure 8

Supplementary Table 1

Supplementary Table 2
