# Correction: Identification of a novel polymorphism associated with reduced clozapine concentration in schizophrenia patients—a genome-wide association study adjusting for smoking habits

**DOI:** 10.1038/s41398-020-01061-4

**Published:** 2020-11-02

**Authors:** Robert Løvsletten Smith, Kevin O’Connell, Lavinia Athanasiu, Srdjan Djurovic, Marianne Kristiansen Kringen, Ole A. Andreassen, Espen Molden

**Affiliations:** 1 Center for Psychopharmacology, Diakonhjemmet Hospital, Oslo, Norway; 2 CoE NORMENT, Division of Mental Health and Addiction, Oslo University Hospital, and Institute of Clinical Medicine, University of Oslo, Oslo, Norway; 3 Department of Medical Genetics, Oslo University Hospital, Oslo, Norway; 4 NORMENT, Department of Clinical Science, University of Bergen, Bergen, Norway; 5 Department of Life Sciences and Health, Oslo Metropolitan University, Oslo, Norway; 6 Department of Pharmacy, Section for Pharmacology and Pharmaceutical Biosciences, University of Oslo, Oslo, Norway

Correction to: *Translational Psychiatry*

10.1038/s41398-020-00888-1 published online 19 June 2020

The authors of the original Article detected typos in Table 2 shortly after publication. This Correction Notice is to confirm that those typos have been corrected.

The typos are as follows:

- In Table 2, p 6, row #10; rs1670747 has been corrected to rs7670472 (wrong lead SNP indicated).

- In table 2, p 6, row #12; rs292638 has been corrected to rs2926038 (a zero was missing between 6 and 3).

- In Table 2, p 6, row #12; ‘G/A has been corrected to ‘A/G G' (wrong effect allele indicated).

