# Supplementary material for: Identification of a novel polymorphism associated with reduced clozapine concentration in schizophrenia patients—a genome-wide association study adjusting for smoking habits
Source: Transl Psychiatry. 2020 Jun 19;10:198. doi: 10.1038/s41398-020-00888-1 (PMC7303159; doi:10.1038/s41398-020-00888-1)
Supplement: Supplementary file 1 — Supplementary methods [file 41398_2020_888_MOESM1_ESM.docx]

**Supplementary methods**

*Meta-analyses for serum concentrations without adjusting for smoking*

We performed meta-analyses of the previously published British GWAS^1^ and each of our corresponding GWASs, without adjusting for smoking. The meta-analyses were performed using the inverse variance-weighted fixed effects models implemented in METAL (http://csg.sph.umich.edu//abecasis/Metal)^2^.

*GWAS of serum concentrations, adjusting for polygenic score proxy measures*

We calculated polygenic scores for body mass index and cigarettes-per-day for all patients following the procedure described in the previously published British GWAS^1^. Briefly, the latest publically available GWAS for body mass index (BMI)^3^ and cigarettes-per-day^4^ were used to generate polygenic scores for all patients. We applied thresholds of GWAS p < 0.05 and LD r2 < 0.1, and generated the polygenic scores using PRSice-2^5^. These values were included as additional covariates in the regression models. This analysis produced similar results to the GWASs without adjusting for smoking (Supplementary Figures 7 and 8, Supplementary Table 2). No loss or gain in genome-wide significant signals was observed for any of the phenotypes.

*Locus definition and annotation*

Independent genomic loci were defined according to the FUMA protocol^6^. First, independent significant SNPs were identified as those with association p-value < 5 x 10^-8^ and linkage disequilibrium (LD) r^2^ < 0.6 with each other. Those with lowest association p-values among these and (LD) r^2^ < 0.1 with each other were then selected as lead SNPs. The borders of the genomic loci were then defined by identifying all candidate SNPs in LD (r^2^ ≤ 0.6) with a lead SNP. Two loci were merged if they were separated by less than 250 kb. The resulting distinct regions were then considered to be single independent genomic loci. All LD information was calculated from the 1000 Genomes Project reference panel^7^.

Positional and functional annotation of all candidate SNPs within each significant genomic locus was performed using ANNOVAR^8^, as implemented in FUMA^6^. SNPs were also annotated with Combined Annotation Dependent Depletion (CADD)^9^ scores, which predict how deleterious the SNP effect is on protein structure/function, RegulomeDB^10^ scores, which predict the likelihood of regulatory functionality, and chromatin states, which predict transcription/regulatory effects from chromatin states at the SNP locus^11, 12^. We also identified previously reported GWAS associations in the NHGRI-EBI catalog^13^ overlapping with the identified loci. Finally, we queried SNPs for known expression quantitative trait loci (eQTLs) in the genotype tissue expression (GTEx) portal^14^.

*Proportion of Variance Explained*

We calculated the proportion of variance explained (PVE) by significant SNPs following the procedure described in the previously published British GWAS^1^, using the PVE method developed by Shim *et al.*^15^.

*Taqman-based targeted SNP genotyping*

The genome-wide significant *NFIB* rs28379954 imputed variant was considered for confirmation by Taqman-based targeted SNP genotyping using a predesigned Taqman SNP Genotyping Assay (C__59359617_10, Thermo Fisher Scientific, Massachusetts, USA). The assay, sample DNA and 2x Taqman Genotyping Master Mix was combined in 8µL reactions and ran on a QuantStudio12K Flex Real-Time PCR System (Thermo Fisher Scientific) using standard conditions. Imputed genotypes for all samples were confirmed by the Taqman genotyping assay.

**References**

1. Pardinas AF et al. Pharmacogenomic Variants and Drug Interactions Identified Through the Genetic Analysis of Clozapine Metabolism. *Am J Psychiatry*. **176**, 477-486 (2019).

2. Willer CJ, Li Y, Abecasis GR. METAL: fast and efficient meta-analysis of genomewide association scans. *Bioinformatics*. **26**, 2190-2191 (2010).

3. Locke AE et al. Genetic studies of body mass index yield new insights for obesity biology. *Nature*. **518**, 197-206 (2015).

4. Consortium TaG. Genome-wide meta-analyses identify multiple loci associated with smoking behavior. *Nat Genet*. **42**, 441-447 (2010).

5. Choi SW, O'Reilly PF. PRSice-2: Polygenic Risk Score software for biobank-scale data. *Gigascience*. **8**, (2019).

6. Watanabe K, Taskesen E, van Bochoven A, Posthuma D. Functional mapping and annotation of genetic associations with FUMA. *Nat Commun*. **8**, 1826 (2017).

7. Auton A et al. A global reference for human genetic variation. *Nature*. **526**, 68-74 (2015).

8. Wang K, Li M, Hakonarson H. ANNOVAR: functional annotation of genetic variants from high-throughput sequencing data. *Nucleic Acids Res*. **38**, e164 (2010).

9. Kircher M et al. A general framework for estimating the relative pathogenicity of human genetic variants. *Nat Genet*. **46**, 310-315 (2014).

10. Boyle AP et al. Annotation of functional variation in personal genomes using RegulomeDB. *Genome Res*. **22**, 1790-1797 (2012).

11. Kundaje A et al. Integrative analysis of 111 reference human epigenomes. *Nature*. **518**, 317-330 (2015).

12. Zhu Z et al. Integration of summary data from GWAS and eQTL studies predicts complex trait gene targets. *Nat Genet*. **48**, 481-487 (2016).

13. MacArthur J et al. The new NHGRI-EBI Catalog of published genome-wide association studies (GWAS Catalog). *Nucleic Acids Res*. **45**, D896-901 (2017).

14. Couchman L, Bowskill SV, Handley S, Patel MX, Flanagan RJ. Plasma clozapine and norclozapine in relation to prescribed dose and other factors in patients aged <18 years: data from a therapeutic drug monitoring service, 1994-2010. *Early Interv Psychiatry*. **7**, 122-130 (2013).

15. Shim H et al. A multivariate genome-wide association analysis of 10 LDL subfractions, and their response to statin treatment, in 1868 Caucasians. *PLoS One*. **10**, e0120758 (2015).
