# Supplementary material for: Identification of a novel polymorphism associated with reduced clozapine concentration in schizophrenia patients—a genome-wide association study adjusting for smoking habits
Source: Transl Psychiatry. 2020 Jun 19;10:198. doi: 10.1038/s41398-020-00888-1 (PMC7303159; doi:10.1038/s41398-020-00888-1)
Supplement: Supplementary file 2 — Supplementary figure and table legends [file 41398_2020_888_MOESM2_ESM.docx]

**Supplementary figure and table legends**

**Supplementary Figure 1.** Meta-analysis of common genetic variants associated with (A) CLZ serum concentration (B) *N-*desmethylclozapine serum concentration and (C) their metabolic ratio, without controlling for smoking habits. Manhattan plots showing the –log10 transformed p-values for each SNP on the y-axis and chromosomal positions along the x-axis. The dotted horizontal line represents the threshold for significant associations (p<5x10^-8^). Independent lead SNPs are encircled in black, and are annotated to the nearest gene.

**Supplementary Figure 2.** QQ plots of the (A) CLZ serum concentration (B) *N*-desmethylclozapine serum concentration and (C) the metabolic ratio GWAS meta-analyses, without controlling for smoking habits.

**Supplementary Figure 3.** Common genetic variants associated with (A) CLZ serum concentration (B) *N-*desmethylclozapine serum concentration and (C) their metabolic ratio, controlling for polygenic score proxy measures. Manhattan plots showing the –log10 transformed p-values for each SNP on the y-axis and chromosomal positions along the x-axis. The dotted horizontal line represents the threshold for significant associations (p<5x10^-8^). Independent lead SNPs are encircled in black, and are annotated to the nearest gene.

**Supplementary Figure 4.** QQ plots of the (A) CLZ serum concentration (B) *N*-desmethylclozapine serum concentration and (C) the metabolic ratio GWAS, controlling for polygenic score proxy measures.

**Supplementary Figure 5.** QQ plots of the (A) CLZ serum concentration (B) *N*-desmethylclozapine serum concentration and (C) the metabolic ratio GWAS analyses, after controlling for smoking habits.

**Supplementary Figure 6**. An overview of the linkage-disequilibrium characteristics of the region on chromosome 9 surrounding rs28379954, within the 1000 genomes British in England and Scotland (GBR) population. Similar results are observed for other European 1000 genomes populations (<http://grch37.ensembl.org/Homo_sapiens/Variation/HighLD?db=core;r=9:14163407-14164407;v=rs28379954;vdb=variation;vf=368074724>). The position of rs28379954 is indicated in green.

**Supplementary Figure 7.** LocusZoom plot for the lead SNP rs28379954. SNPs in LD (r^2^ > 0.2) with rs28379954 are also labelled. All SNPs flanking the lead SNP (within 250kb) are plotted. Linkage disequilibrium values (r^2^) between the lead SNP and all other SNPs within the region are shown. Recombination rates for the plotted region are shown.

**Supplementary Figure 8.** QQ plots of the (A) CLZ serum concentration (B) *N*-desmethylclozapine serum concentration and (C) the metabolic ratio GWAS analyses, without controlling for smoking habits.

**Supplementary table 1.** Association statistics and effect sizes of the lead SNPs for each phenotype and genomic locus from the meta-analyses.

**Supplementary table 2.** Association statistics and effect sizes of the lead SNPs for each phenotype and genomic locus from GWAS, after controlling for polygenic score proxy measures.
