# Supplementary figures and images for: Identification of a novel polymorphism associated with reduced clozapine concentration in schizophrenia patients—a genome-wide association study adjusting for smoking habits
Source: Transl Psychiatry. 2020 Jun 19;10:198. doi: 10.1038/s41398-020-00888-1 (PMC7303159; doi:10.1038/s41398-020-00888-1)

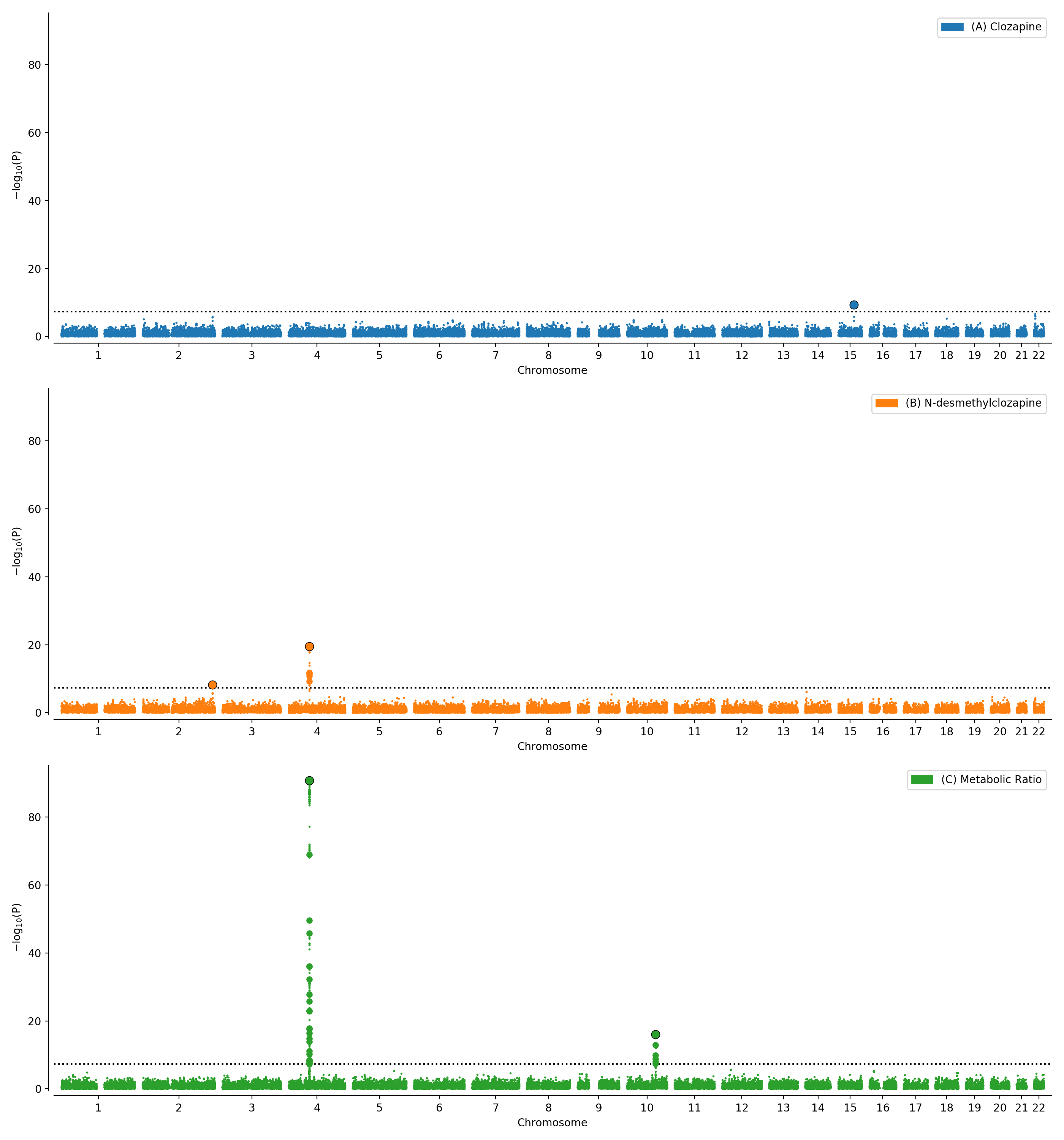

Supplement: Supplementary file 3 — Supplementary figure 1 [file 41398_2020_888_MOESM3_ESM.png]

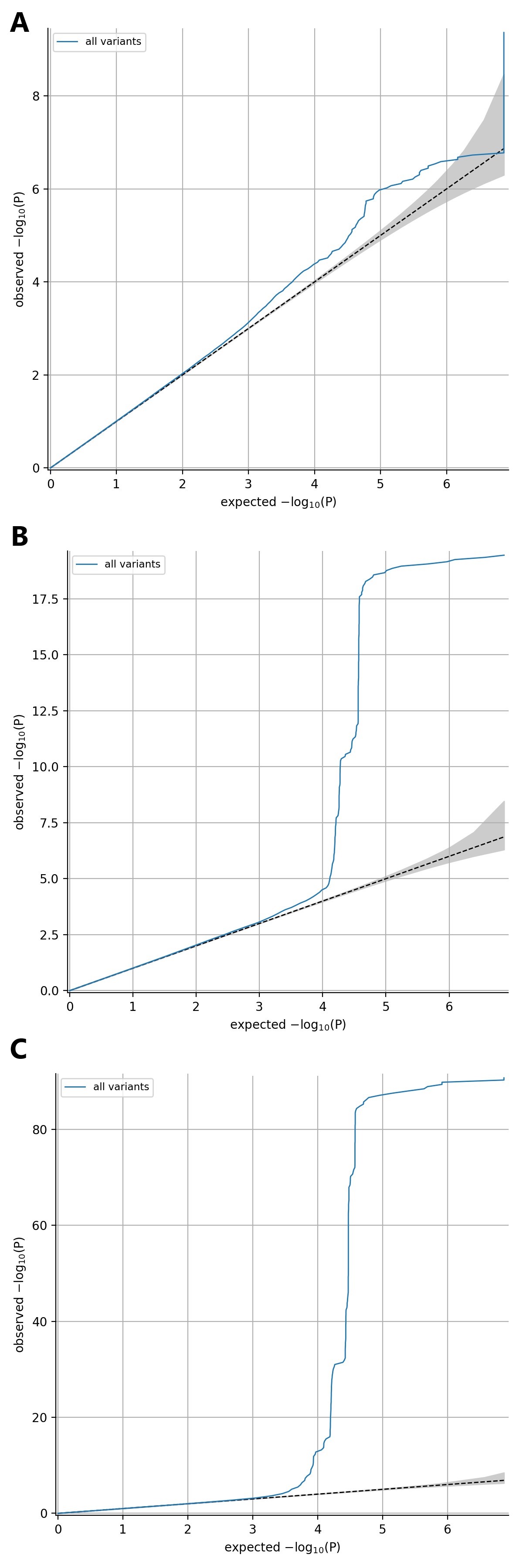

Supplement: Supplementary file 4 — Supplementary figure 2 [file 41398_2020_888_MOESM4_ESM.jpg]

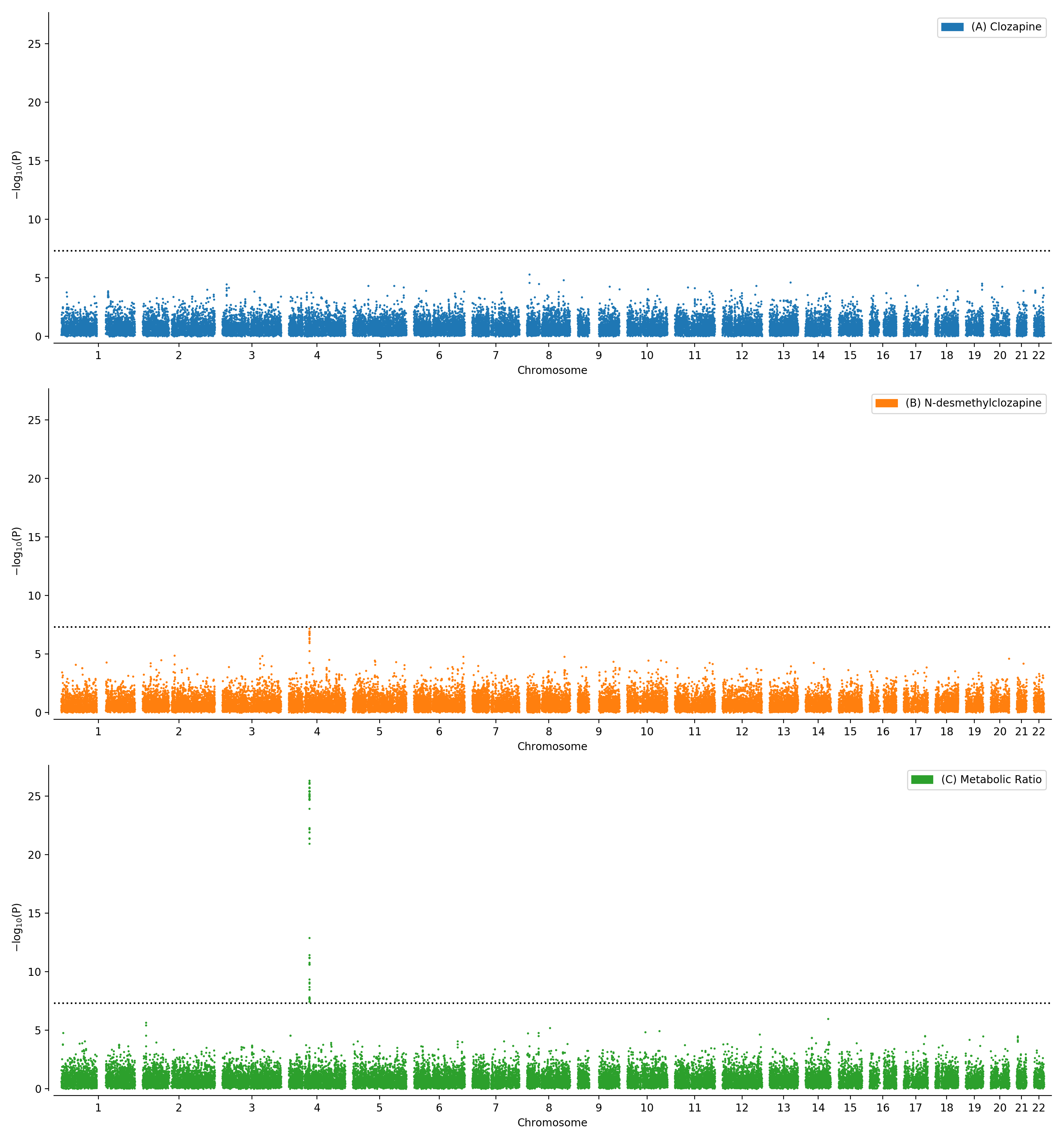

Supplement: Supplementary file 5 — Supplementary figure 3 [file 41398_2020_888_MOESM5_ESM.png]

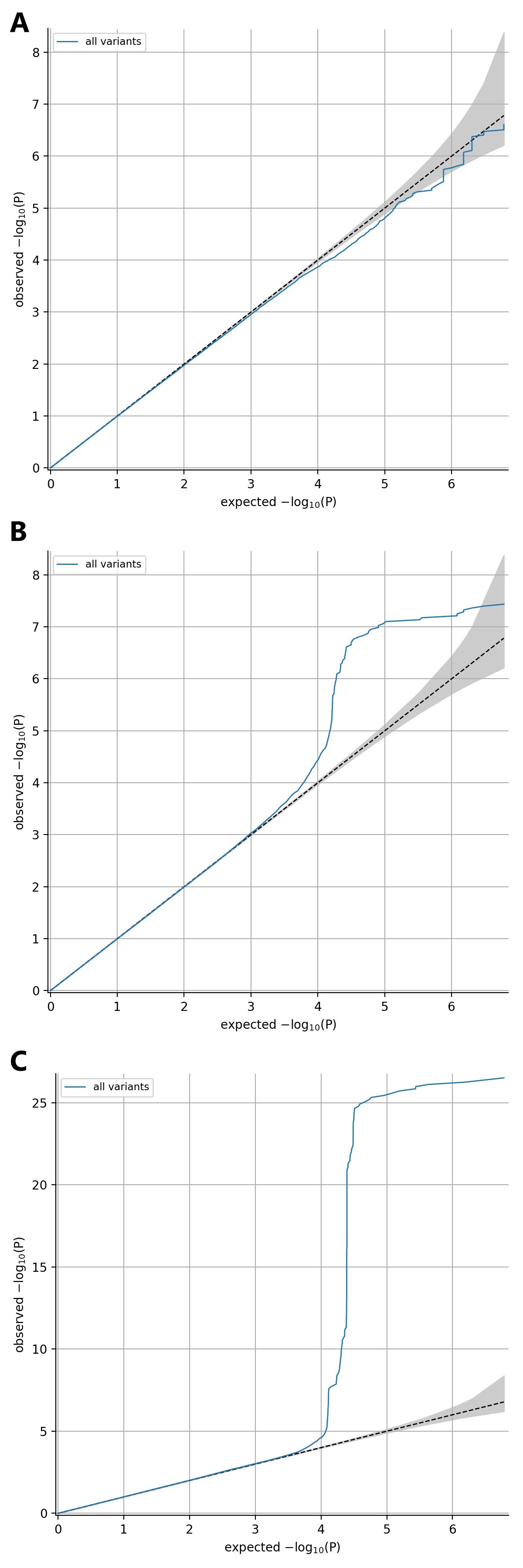

Supplement: Supplementary file 6 — Supplementary figure 4 [file 41398_2020_888_MOESM6_ESM.jpg]

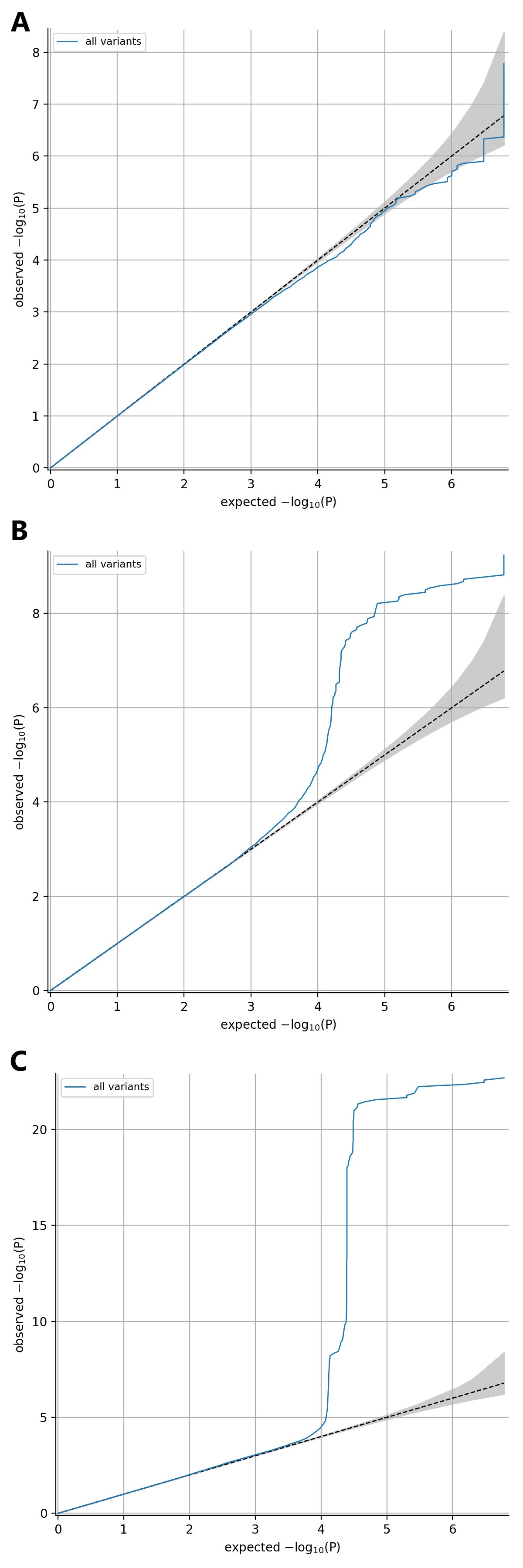

Supplement: Supplementary file 7 — Supplementary figure 5 [file 41398_2020_888_MOESM7_ESM.jpg]

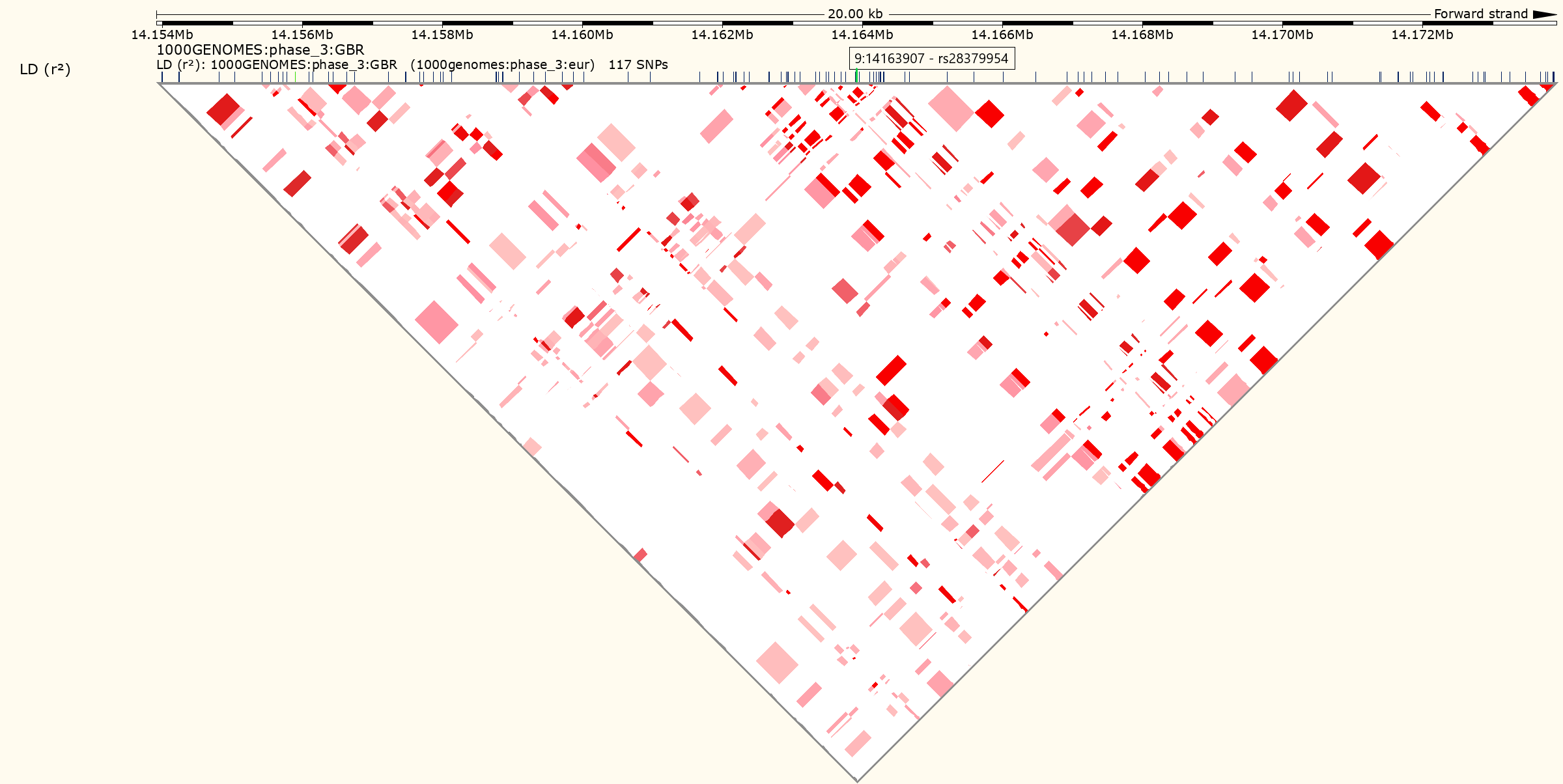

Supplement: Supplementary file 8 — Supplementary figure 6 [file 41398_2020_888_MOESM8_ESM.png]

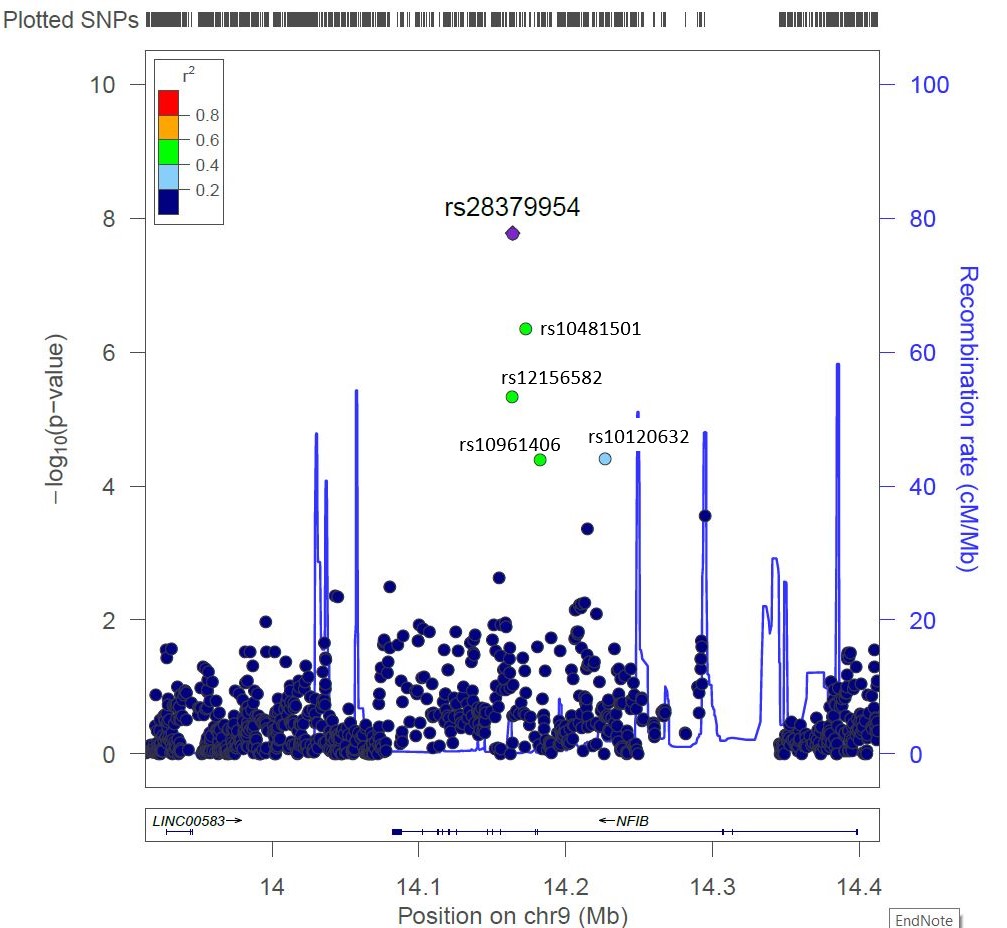

Supplement: Supplementary file 9 — Supplementary figure 7 [file 41398_2020_888_MOESM9_ESM.jpg]

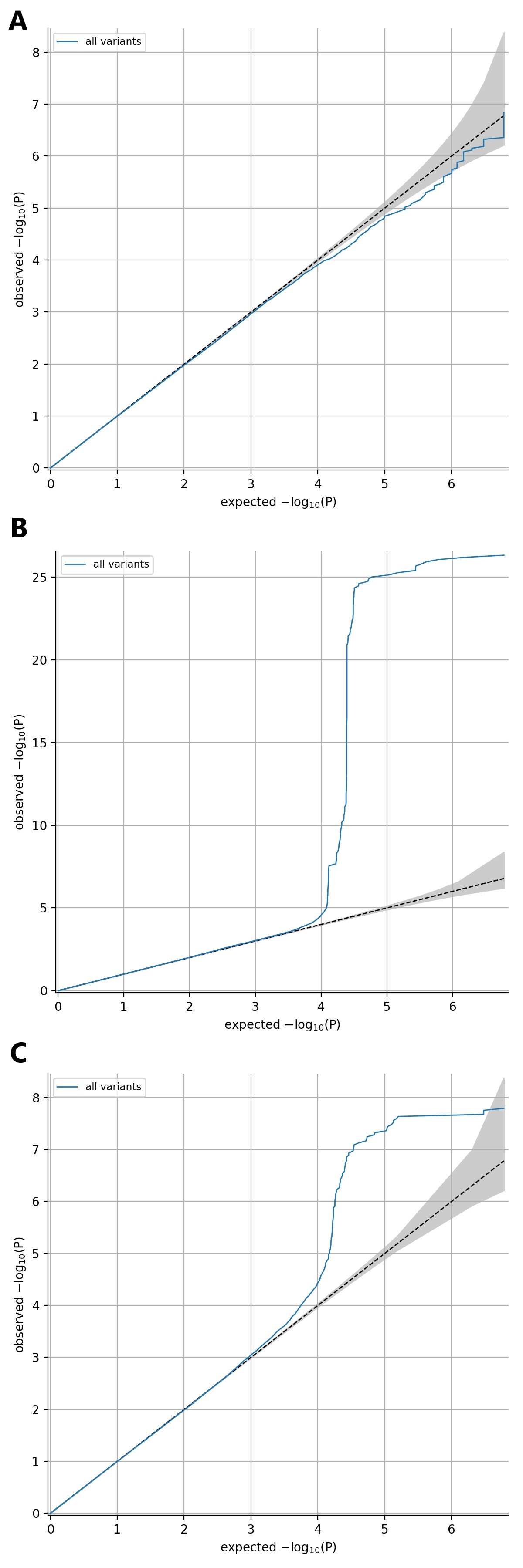

Supplement: Supplementary file 10 — Supplementary figure 8 [file 41398_2020_888_MOESM10_ESM.jpg]
