# Supplementary material for: Identification of a novel polymorphism associated with reduced clozapine concentration in schizophrenia patients—a genome-wide association study adjusting for smoking habits
Source: Transl Psychiatry. 2020 Jun 19;10:198. doi: 10.1038/s41398-020-00888-1 (PMC7303159; doi:10.1038/s41398-020-00888-1)
Supplement: Supplementary file 11 — Supplementary Table 1 [file 41398_2020_888_MOESM11_ESM.docx]

| **Phenotype and Locus** | **Lead SNP** | **Position: Alleles** | **Effect Allele** | **GWAS p** | **Beta** | **SE** | **Annotation** | **Nearest Gene** | **Dist** | **CADD** | **RDB** | **Min**  **Chr**  **State** | **Common**  **Chr**  **State** |
| --- | --- | --- | --- | --- | --- | --- | --- | --- | --- | --- | --- | --- | --- |
| Clozapine: |  |  |  |  |  |  |  |  |  |  |  |  |  |
| 15: 74817689 - 75027880 | rs2472297 | 75027880: C/T | T | 4.345 x 10^-10^ | 0.073 | 0.012 | Intergenic | *CYP1A1* | 9928 | 0.604 | 4 | 5 | 14 |
| *N*-desmethylclozapine: | |  |  |  |  |  |  |  |  |  |  |  |  |
| 2: 234611523 - 234673588 | rs3732218 | 234627304: G/A | A | 7.381 x 10^-9^ | 0.095 | 0.016 | Intronic | *UGT1A5, UGT1A6, UGT1A7, UGT1A8, UGT1A9, UGT1A10* | 0  0  0  0  0  0 | 2.199 | NA | 1 | 15 |
| 4: 69601886 - 70138176 | rs10000284 | 69669183: C/T | T | 3.553 x 10^-20^ | -0.140 | 0.015 | Intergenic | *RP11-468N14.1* | 10529 | 0.147 | 6 | 7 | 15 |
| Metabolic Ratio: |  |  |  |  |  |  |  |  |  |  |  |  |  |
| 4: 69535335 - 70507545 | rs10023464 | 69659738: C/T | T | 1.770 x 10^-91^ | -0.177 | 0.009 | Intergenic | *CTD-2005D20.1* | 6545 | 0.989 | 7 | 14 | 15 |
| 10: 96131661 - 96851615 | rs12767583 | 96547463: C/T | T | 9.865 x 10^-17^ | -0.063 | 0.008 | Intronic | *CYP2C19* | 0 | 0.553 | 7 | 4 | 15 |
| CADD = Combined Annotation-Dependent depletion score, which predict how deleterious the SNP effect is on protein structure/function (higher scores indicate more deleterious); RegulomeDB (RDB) scores predict likelihood of regulatory functionality (lower scores indicate higher likelihood); minChrState = minimum chromatin state across 127 tissue types (lower scores indicate more open chromatin); commonChrState = most common chromatin state in 127 tissue types. | | | | | | | | | | | | | |
