# Supplementary material for: Identification of a novel polymorphism associated with reduced clozapine concentration in schizophrenia patients—a genome-wide association study adjusting for smoking habits
Source: Transl Psychiatry. 2020 Jun 19;10:198. doi: 10.1038/s41398-020-00888-1 (PMC7303159; doi:10.1038/s41398-020-00888-1)
Supplement: Supplementary file 12 — Supplementary Table 2 [file 41398_2020_888_MOESM12_ESM.docx]

| **Phenotype and Locus** | **Lead SNP** | **Position: Alleles** | **Effect Allele** | **GWAS p** | **Beta** | **SE** | **Annotation** | **Nearest Gene** | **Dist** | **CADD** | **RDB** | **Min**  **Chr**  **State** | **Common**  **Chr**  **State** |
| --- | --- | --- | --- | --- | --- | --- | --- | --- | --- | --- | --- | --- | --- |
| *N*-desmethylclozapine: | |  |  |  |  |  |  |  |  |  |  |  |  |
| 4: 69601886 - 70138176 | rs10028938 | 69669216: A/G | A | 3.669 x  10^-8^ | -0.260 | 0.046 | Intergenic | *RP11-468N14.1* | 10496 | 1.153 | 7 | 7 | 15 |
| Metabolic Ratio: | |  |  |  |  |  |  |  |  |  |  |  |  |
| 4: 69535335 - 70387482 | rs1513559 | 69655555: A/G | G | 3.083 x 10^-27^ | 0.246 | 0.021 | Intergenic | *CTD-2005D20.1* | 2362 | 0.004 | 5 | 9 | 15 |
| CADD=Combined Annotation-Dependent depletion score, which predict how deleterious the SNP effect is on protein structure/function (higher scores indicate more deleterious); RegulomeDB (RDB) scores predict likelihood of regulatory functionality (lower scores indicate higher likelihood); minChrState=minimum chromatin state across 127 tissue types (lower scores indicate more open chromatin); commonChrState=most common chromatin state in 127 tissue types. | | | | | | | | | | | | | |
